# A study of the effects of screen exposure on the neuropsychological development in children with autism spectrum disorders based on ScreenQ

**DOI:** 10.1186/s12887-024-04814-y

**Published:** 2024-05-16

**Authors:** Xinyue Peng, Yang Xue, Hanyu Dong, Chi Ma, Feiyong Jia, Lin Du

**Affiliations:** 1 Department of Developmental and Behavioral Pediatrics, Children’s Medical Center, The First Hospital of Jilin University, Changchun, 130021 China; 2 School of Nursing, Jilin University, Changchun, 130021 China

**Keywords:** Screen exposure, CARS, GDS-C, Development quotient

## Abstract

**Purpose:**

To investigate the relationship between multi-dimensional aspects of screen exposure and autistic symptoms, as well as neuropsychological development in children with ASD.

**Methods:**

We compared the ScreenQ and Griffiths Development Scales-Chinese Language Edition (GDS-C) of 636 ASD children (40.79 ± 11.45 months) and 43 typically developing (TD) children (42.44 ± 9.61 months). Then, we analyzed the correlations between ScreenQ and Childhood Autism Rating Scale (CARS), and GDS-C. We further used linear regression model to analyze the risk factors associated with high CARS total scores and low development quotients (DQs) in children with ASD.

**Results:**

The CARS of children with ASD was positively correlated with the ScreenQ total scores and “access, frequency, co-viewing” items of ScreenQ. The personal social skills DQ was negatively correlated with the “access, frequency, content, co-viewing and total scores” of ScreenQ. The hearing-speech DQ was negatively correlated with the “frequency, content, co-viewing and total scores” of ScreenQ. The eye-hand coordination DQ was negatively correlated with the “frequency and total scores” of ScreenQ. The performance DQ was negatively correlated with the “frequency” item of ScreenQ.

**Conclusion:**

ScreenQ can be used in the study of screen exposure in children with ASD. The higher the ScreenQ scores, the more severe the autistic symptoms tend to be, and the more delayed the development of children with ASD in the domains of personal-social, hearing-speech and eye-hand coordination. In addition, “frequency” has the greatest impact on the domains of personal social skills, hearing-speech, eye-hand coordination and performance of children with ASD.

## Introduction

Autism Spectrum Disorder (ASD) is a neurodevelopmental disorder characterized by challenges in social interaction, communication (both verbal and nonverbal), limited interests, and repetitive activities [1]. The prevalence of autism is 1 in 36, with a progressive trend over time [2]. The life-long impact of ASD imposes a significant economic burden on individuals, families, and society [3].

The cause of ASD remains uncertain, and it may stem from the combined influence of environmental and genetic factors [4]. In recent years, media devices have become ubiquitous among children, with a gradual increase in children’s electronic screen exposure. The vast majority of children first encounter screens before the age of two [5]. Children had an average of 53 min of screen time at the age of 1 and by 3 years, they had more than 150 min [6]. In the early development of children with ASD, an important environmental factor is electronic screen exposure [7]. The American Academy of Pediatrics (AAP) advises against exposing toddlers and infants under the age of 18 to 24 months to any digital media and advises against their using media on their own. It is advised that children between the ages of two and five spend no more than one hour a day using screens. Parents should cover with their kids, aid them in comprehending what they are seeing and in applying their acquired knowledge to their surroundings [8]. However, research indicates that 40 to 50% of children between the ages of 3 and 6 use screens for more than two hours per day [9, 10]. Especially in recent years with the outbreak of COVID-19, the percentage of preschool children who use screens for more than two hours a day has risen to 84.1% [11]. The escalating rise in children’s screen exposure is more and more serious. According to a Chinese cohort study, early exposure to screens was linked to poor cognitive and social-emotional development [12]. Another cohort study of 3895 children aged 1 to 3 years clustered children’s screen time trajectories into 2 distinct patterns of stable low vs. increasing use (26.7%). Increasing trajectory status was associated with an additional 15 to 20 min of daily screen time at 7 to 8 years of age [6].

Compared to typically developing children, ASD children tended to present an earlier age of exposure to screen devices and have longer screen exposure (3.34 ± 2.64 h vs.0.91 ± 0.93 h) [13], even more than 3 h per day [14]. Some study suggested that the children who used electronics more frequently were far more likely to experience behavioral issues and symptoms similar to autism spectrum disorder [11, 15]. Longer screen time at 1 year of age was substantially related with an ASD diagnosis at 3 years of age, according to a Japanese study that looked at the relationship between screen time exposure in children at 1 year of age and ASD at 3 years of age [16]. Therefore, the impact of screen exposure on children’s growth and development should be carefully considered.

Most of the measures used in previous studies have been inconsistent [17], mostly involving single frequency items for television, smart phones, or other media, and lacking comprehensive measures of screen exposure for children with ASD. Most current studies on screen exposure are based on screen time, and there is a lack of in-depth exploration of the frequency, content, etc. that are closely related to screen-based media use. In order to quantify screen-based media consumption in a group of preschool-aged children with ASD and TD, this study used a comprehensive, parent-reported measure (ScreenQ) to reflect multi-dimensional aspects of use, including access to screens, frequency of use, media content, and caregiver–child co-viewing. The development of ScreenQ was to reflect AAP recommendations on media use [8] and the items are summed for a total score from 0 to 27. A score of 0 reflects perfect adherence to AAP recommendations, and higher scores reflect greater use contrary to recommendations. This instrument was used to offset the shortcomings of previous studies that investigated screen exposure in children with ASD [17, 18]. Meanwhile, the purpose of this study was to investigate the effects of access to screens, frequency of screen use, content viewed, and caregiver–child co-viewing on neuropsychological development in preschool children with ASD.

## Methods

### Participants

636 children (490 boys and 146 girls) diagnosed with ASD for the first time in the Department of Developmental and Behavioral Pediatrics of the First Hospital of Jilin University between January 2021 and December 2022 were chosen as the ASD group. The age of the ASD children was 40.79 ± 11.45 months. During the same period, 43 TD children (32 boys and 11 girls), matched for gender and age (42.44 ± 9.61 months), who underwent routine physical examinations in the Department of Developmental and Behavioral Pediatrics were chosen as the TD group. The inclusion criterion for children with ASD: the diagnosis of ASD was determined using the DSM-5 and the Autism Diagnostic Observation Schedule–Second Edition (ADOS-2). The children with ASD didn’t have genetic disorders. The inclusion criteria for children with TD: healthy children who receive regular physical examinations and they have not been diagnosed with neurodevelopmental disorders. Children with severe physical disability, cardiopulmonary disease, epilepsy were not included. The current research was confirmed by the Hospital’s institutional ethics committee. This work was supported by the National Natural Science Foundation of China (Grant number: 81,973,054), Youth Development Fund of the First Hospital of Jilin University (Grant number: JDYY14202328) and the health science and technology ability improvement project of Jilin Province (Grant number: 2023LC005).

### Procedure

We collected the general information, including name, sex, age, place of residence, parents’ education levels from two groups. All parents completed the ScreenQ. The Griffiths Development Scales-Chinese Language Edition (GDS-C) and the Childhood Autism Rating Scale (CARS) were used to examine the developmental level and symptoms of ASD in the ASD group. The children in the TD group were examined using only the GDS-C. We compared the general information, ScreenQ scores and the development quotients (DQs) of all domains of the GDS-C of the ASD group and the TD group. Then, we analyzed the correlations between ScreenQ scores and CARS scores, GDS-C in the ASD group. We further used linear regression model to analyze the risk factors associated with higher CARS total scores, lower DQs in children with ASD.

Systematically trained assessors conducted all scale assessments and developmental tests with high consistency. The assessors were not involved in the study design and were unaware of the grouping and purpose of the study.

The ADOS-2 is a semi-structured, standardized observational assessment that is considered the gold standard for diagnosing ASD [19]. Its purpose is to assess social-communicative behaviors alongside stereotypical and repetitive behavioral features. Assessors rate children on items linked to social affect (SA) and restricted and repetitive behaviors (RRB) during the evaluation, based on their interactions and interviews with children. Higher scores indicate more severe intensity [20].

**Fig. 1:**
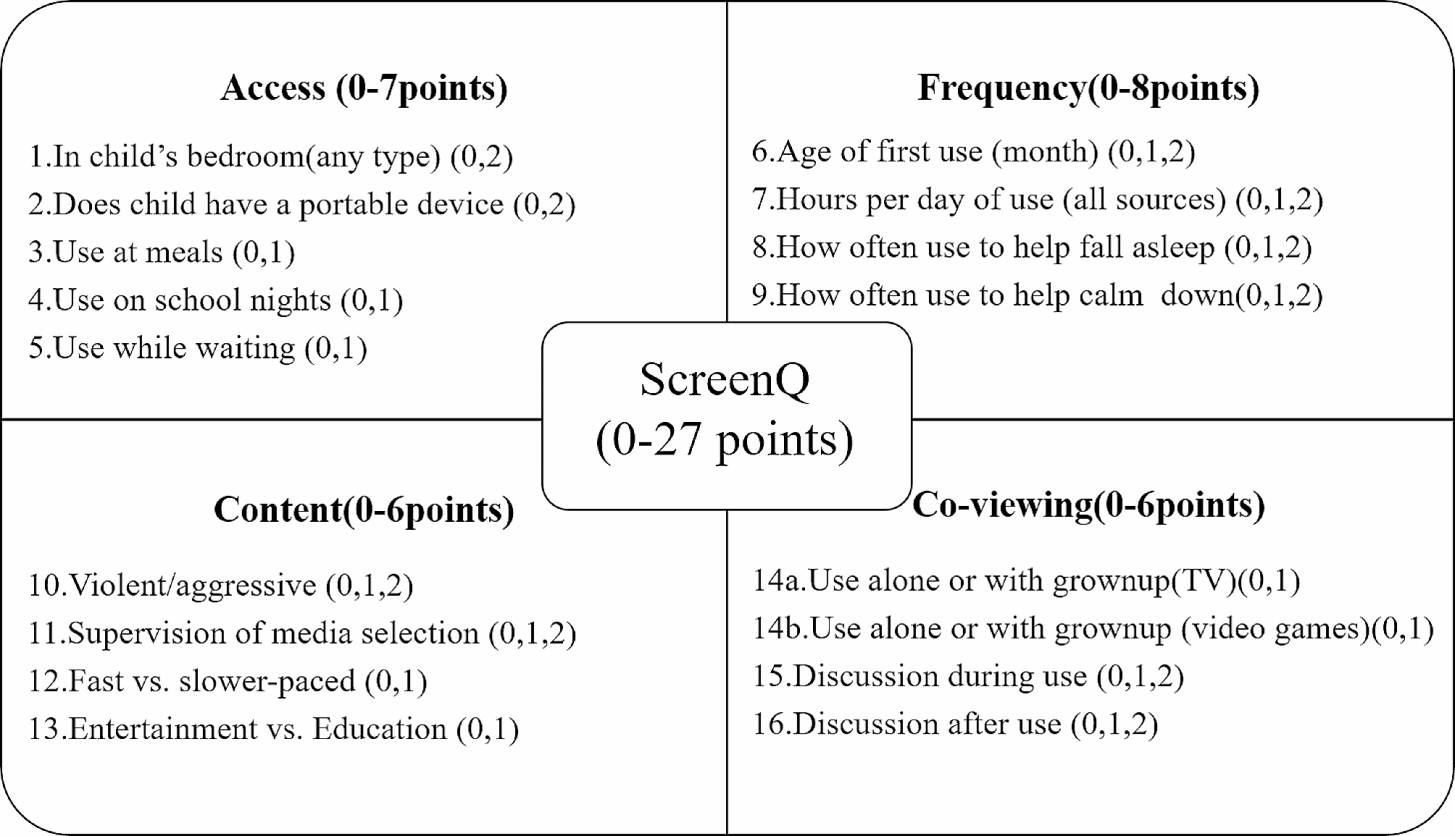
Conceptual diagram of the ScreenQ measure and its four domains

The ScreenQ is a 16-item composite measure of screen-based media use of children, reflecting the domains in the AAP recommendations: access to screens, frequency of use, media content and caregiver–child co-viewing [21]. The 16 items are summarized in Fig. 1. The ScreenQ has strong internal consistency (Cronbach α = 0.74) [22] and validity [18]. The range of scores for ScreenQ is 0–27. A score of 0, which represents perfect compliance with AAP recommendations. On the contrary, higher scores indicate a greater usage than the AAP recommendations.

The GDS-C is a valid and reliable neurodevelopmental scale used to assess the neuropsychological development of children aged 0–8 years with good reliability and validity in China [23, 24]. The GDS-C includes 5 domains: physical mobility, personal social skills, hearing-speech, eye-hand coordination and performance. The developmental quotient (DQ) is calculated as 100×(developmental age/chronological age). Each domain has a corresponding DQ score and a higher DQ indicates better developmental level in children.

The CARS is a widely used diagnostic scale in China that has strong reliability and validity [25–27]. It consists of 15 items covering children’s social interaction, language, behavior and sensory aspects. The score for each item ranges from normal to severely abnormal. The CARS requires the assessor to observe the behavior of children with ASD in an assessment room. The higher the total score, the more severe the ASD symptoms.

### Statistical methods

The analyses were all carried out using the SPSS 23.0 (Statistical Package for Social Science version 23.0). Categorical variables were represented as frequency (percentage). Chi-square tests were used to measure the differences between the two groups. Continuous variables with normal distribution were represented as the mean ± standard deviation. Two independent-sample t-test was used for the comparisons between the groups. Continuous variables with non-normal distribution were represented by the median (P_25_, P_75_). Two independent-samples Mann-Whitney U test was used for the comparisons between the groups. The Spearman rank correlation analysis was used to analyze the correlations between ScreenQ scores, CARS scores and GDS-C in the ASD group. A linear regression model was used to analyze the risk factors associated with higher CARS total scores, lower personal social skills DQ and hearing-speech DQ in ASD children. *P* < 0.05 was considered to be statistically significant.

## Result

### General information, GDS-C, and screen Q scores in the ASD group and TD group

There were no differences in sex (*P* = 0.693), age (*P* = 0.356), place of residence (*P* = 0.748), maternal education (*P* = 0.203) and paternal education (*P* = 0.341) between ASD group and TD group. The development quotients in the ASD group were significantly lower than that in the TD group (*P* < 0.001). The ScreenQ scores in the ASD group were significantly higher than those in the TD group (*P* < 0.001) (Table 1).

**Table 1:** Comparison of general information, GDS-C and screenQ in two groups

Group	ASD group *N* = 636	TD group *N* = 43	t/${\varvec{\chi }}^{2}$/Z	*P*
Sex, %			0.156	0.693
Male	490(77.0%)	32(74.4%)		
Female	146(23.0%)	11(25.6%)		
Age(m), (mean ± SD)	40.79 ± 11.45	42.44 ± 9.61	0.923	0.356
Place of residence, %			0.580	0.748
Urban	439(69.0%)	32(74.4%)		
Township	84(13.2%)	5(11.6%)		
Rural	113(17.8%)	6(14.0%)		
Maternal education, %			1.621	0.203
Senior high school or below	330(51.9%)	18(41.9%)		
Junior college or above	306(48.1%)	25(58.1%)		
Paternal education, %			0.907	0.341
Senior high school or below	358(56.3%)	21(48.8%)		
Junior college or above	278(43.7%)	22(51.2%)		
GDS-C(DQ), M(P_25_,P_75_)				
physical mobility	69(59,81)	100(75,113)	-7.137	< 0.001^*^
personal social skills	51(40,64.75)	100(90,109)	-10.249	< 0.001^*^
hearing-speech	37(26,55)	88(71,103)	-9.900	< 0.001^*^
eye-hand coordination	58(45,71)	100(89,103)	-10.131	< 0.001^*^
performance	63(47,78)	100(92,114)	-9.119	< 0.001^*^
ScreenQ, M(P_25_,P_75_)				
access	6 (2, 6)	1(0,2)	-9.652	< 0.001^*^
frequency	2 (2, 4)	1 (1, 2)	-5.861	< 0.001^*^
content	2 (2)	1(0,1)	-8.383	< 0.001^*^
co-viewing	5 (2, 5)	2(0,3)	-7.930	< 0.001^*^
total scores	14 (11, 17)	5 (3, 6)	-9.778	< 0.001^*^

^*^P < 0.05

### Correlations between screenQ scores and CARS scores in children with ASD

**Fig. 2:**
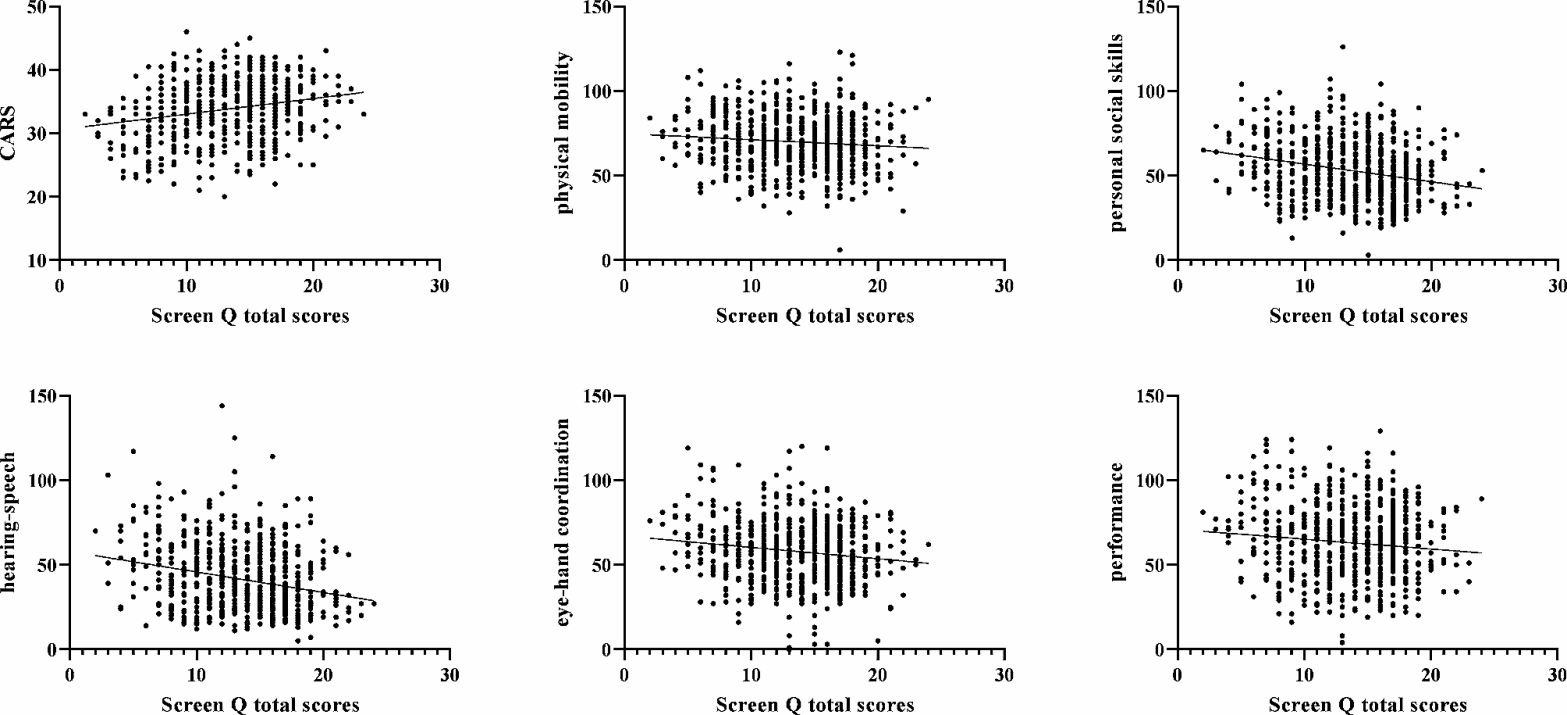
Correlation between ScreenQ and CARS and the DQs of all domains of the GDS-C

The results of the Spearman rank correlation test showed that total score of CARS of ASD children was positively correlated with the total score of ScreenQ (*r* = 0.201, *P* < 0.001) and “access (r = 0.103, *P* = 0.010), frequency (r = 0.195, *P* < 0.001), co-viewing (r = 0.205, *P* < 0.001)” item of ScreenQ (Table 2; Fig. 2).

**Table 2:** Correlations between screen Q scores and CARS total scores

	Access	Frequency	Content	Co-viewing	ScreenQ total scores
r	0.103	0.195	0.091	0.205	0.201
*P*	0.010^*^	< 0.001^*^	0.022^*^	< 0.001	< 0.001^*^

^*^P < 0.05

### Correlations between screenQ scores and DQs in children with ASD

The personal social skills DQ was negatively correlated with the “access (r = -0.126, *P* = 0.001), frequency (r = -0.219, *P* < 0.001), content (r = -0.155, *P* < 0.001), co-viewing (r = -0.185, *P* < 0.001) and total scores (r = -0.245, *P* < 0.001)” of ScreenQ. The hearing-speech DQ was negatively correlated with the “frequency (r = -0.232, *P* < 0.001), content (r = -0.146, *P* < 0.001), co-viewing (r = -0.178, *P* < 0.001) and total scores (r = -0.225, *P* < 0.001)” of ScreenQ. The eye-hand coordination DQ was negatively correlated with the “frequency (r = -0.145, *P* < 0.001) and total scores (r = -0.130, *P* = 0.001)” of ScreenQ. The performance DQ was negatively correlated with the “frequency (r = -0.103, *P* = 0.010)” item of ScreenQ (Table 3; Fig. 2).

**Table 3:** Correlations between screenQ scores and DQs in the ASD group

		Access	Frequency	Content	Co-viewing	ScreenQ total scores
physical mobility	r	-0.064	-0.075	-0.090	-0.060	-0.092
	*P*	0.105	0.058	0.023*	0.130	0.020*
personal social skills	r	-0.126	-0.219	-0.155	-0.185	-0.245
	*P*	0.001*	< 0.001*	< 0.001*	< 0.001*	< 0.001*
hearing-speech	r	-0.096	-0.232	-0.146	-0.178	-0.225
	*P*	0.015*	< 0.001*	< 0.001*	< 0.001*	< 0.001*
eye-hand coordination	r	-0.078	-0.145	-0.070	-0.091	-0.130
	*P*	0.050	< 0.001*	0.078	0.022*	0.001*
performance	r	-0.053	-0.103	-0.041	-0.063	-0.093
	*P*	0.182	0.010*	0.306	0.110	0.036*

^*^P < 0.05

### Linear Regression Results of the Risk Factors for High CARS Total Scores and Low DQs in Children with ASD

The statistically significant factors in the correlation analysis were incorporated into the linear regression model (Tables 2 and 3). The frequency of use (B = 0.143, t = 3.504, *P* < 0.001) and caregiver–child co-viewing (B = 0.176, t = 4.249, *P* < 0.001) are risk factors for high CARS total scores. Caregiver–child co-viewing had the greatest impact on changes in CARS. The frequency of use (B = -0.147, t = -3.612, *P* < 0.001), content view (B = -0.097, t = -2.498, *P* = 0.013) and caregiver–child co-viewing (B = -0.138, t = -3.322, *P* = 0.001) are risk factors for low personal social skills DQ. The frequency of use had the greatest impact on changes in personal social skills DQ. The frequency of use (B = -0.197, t = -5.002, *P* < 0.001) and caregiver–child co-viewing (B = -0.121, t = -3.089, *P* = 0.002) are risk factors for low hearing-speech DQ. The frequency of use had the greatest impact on changes in hearing-speech DQ. The frequency of use (B = -0.138, t = -3.498, *P* = 0.001) is the risk factor for low eye-hand coordination DQ. The frequency of use (B = -0.088, t = -2.233, *P* = 0.026) is the risk factor for low performance DQ (Table 4).

**Table 4:** Linear regression results of the risk factors for CARS total scores, DQs in ASD children

		Access	Frequency	Content	Co-viewing
CARS total scores	B	0.011	0.143	-	0.176
	t	0.264	3.504	-	4.249
	*P*	0.792	< 0.001*	-	< 0.001*
physical mobility	B	-	-	-	-
	t	-	-	-	-
	*P*	-	-	-	-
personal social skills	B	-0.026	-0.147	-0.097	-0.138
	t	-0.613	-3.612	-2.498	-3.322
	*P*	0.540	< 0.001*	0.013*	0.001*
hearing-speech	B	-	-0.197	-0.073	-0.121
	t	-	-5.002	-1.892	-3.089
	*P*	-	< 0.001*	0.059	0.002*
eye-hand coordination	B	-	-0.138	-	-
	t	-	-3.498	-	-
	*P*	-	0.001*	-	-
performance	B	-	-0.088	-	-
	t	-	-2.233	-	-
	*P*	-	0.026*	-	-

^*^P < 0.05

## Discussion

ScreenQ is a novel, composite parent report measure of screenbased media use. Our study is the first to use ScreenQ to analyze the correlation between screen exposure and autistic symptoms, as well as the correlation between screen exposure and neuropsychological development in children with ASD. The main findings of this study are as follows: 1.the ScreenQ scores of children with ASD were significantly higher than those of TD children; 2. the higher the ScreenQ scores, the more severe the autistic symptoms tend to be, and the lower the DQs in children with ASD; 3.frequency of screen use is the risk factor for low DQs.

### ScreenQ was related to CARS total scores in children with ASD

Our results showed that the CARS total scores were positively correlated with the ScreenQ total scores. In our study, higher ScreenQ scores correlate with more pronounced autistic symptoms. Previous studies have shown that the screen time of ASD children is related to their autism symptoms [13], which is consistent with the results of our study. There is a significant association between ASD-like symptoms and the amount of screen time spent on devices [11]. Screen exposure in early life might increase the occurrence of autistic-like behaviors among preschoolers, and excessive screen media exposure may cause symptoms resembling ASD [28, 29].

This study is unique because we are the first to use ScreenQ to analyze the correlation between screen exposure and autistic symptoms. We conducted correlation study on the CARS total scores and the 4 items of the ScreenQ. The findings indicated a positive correlation between the CARS total scores and the “access, frequency, co-viewing” items. In our opinion, children with ASD who spend extended hours with screens, especially without adult facilitation, are more likely to exhibit more severe ASD symptoms. On the contrary, excessive screen exposure can also be seen as a consequence of ASD. The more severe the symptoms of ASD, the higher the ScreenQ scores may be due to reasons such as the strong sensory stimulation needs of children with ASD, parents of children with ASD being more inclined to use electronic devices as contemporary parenting tools, etc.

In addition to the frequency of use as a risk factor, we also found that co-viewing is also a risk factor for high CARS total scores. A nationally representative research conducted in France revealed a complex association between the risk of ASD and screen exposure. Compared to children who never or very seldom used screen media, those who used it on a daily or weekly basis were more likely to be at moderate risk of neurodevelopmental problems [30]. It reflected that screen exposure can affect children’s neurodevelopment to some extent. In addition to having an impact on parent-child interaction, screen exposure also interferes with real-life social interactions [7]. Furthermore, the developmental outcomes of children can be positively impacted by parents who maintain social connections and are actively involved in their children’s lives [31–33].

Therefore, according to our results, children should be assigned a reasonable frequency of use and screen time, choose content that is appropriate for their age and developmental level, avoid overly violent or fast-paced games or programs, and caregivers should make an effort to increase opportunities for parent-child interaction, promote communication, and impart new skills.

### ScreenQ was related to the neuropsychological development of the children with ASD

In this study, the development of personal social skills, hearing-speech and eye-hand coordination in ASD children was related to the ScreenQ total scores. The higher the ScreenQ total scores, the more serious the impact on neuropsychological development in children with ASD. In particular, “frequency” has the greatest impact on personal social DQ, hearing-speech DQ, eye-hand coordination DQ and performance DQ in children with ASD. Also, children with poorer neurological development may lean towards low-interaction, sedentary activities such as electronic screens, leading to higher ScreenQ scores.

Higher access to screens is related to decreased functional connectivity between neural networks associated with basic attention skills and cognitive control in children [34]. In our opinion, the concern is the increase of screen exposure at the expense of activities and social interaction by talking and being in contact with others. Firstly, the increased use of screen-based media was associated with lower microstructural integrity of brain white matter tracts that support language, executive functions, and emergent literacy skills [22]. The longer the screen exposure, the less attention will be paid to parental voice and the less parent-child interaction occurs [35], resulting in less vocalization by the child, which affects language development and the parent-child relationship. Secondly, the longer the sitting time, the less time spent outdoors, leading to possible problems with social skills [36]. Thirdly, television programmes and mobile games usually contain fast-paced, violent and aggressive content which are not developmentally appropriate for pre-school children. In particular, children with ASD experience more frequent and earlier screen time than children with TD [37, 38], so children with ASD may be at more risk for developmental and behavioral problems.

Some studies have some similar results to ours. Screen exposure is negatively associated with DQs in children with ASD [39]. According to a Korean study, there is a direct link between screen time and language delay. Children who watch television for two hours or more are at a 2.7-fold higher risk of language delay, and the risk of language delay rises as toddlers watch more TV [40]. Another study found that screen time affects children’s language skill, and most children had less parent-child interaction during viewing in children with ASD [41].

Sugiyama et al [36] discovered that higher screen usage at age 2 years was connected with poorer speech and daily living abilities but was not associated with socialization. However, another study indicated that screen exposure may be adversely associated with preschool children’s social skills [42]. In our study, the personal social skills DQ was negatively correlated with the total scores of ScreenQ. While the deficit of personal social skills is a core symptom of the autistic condition, it is possible that excessive screen exposure could exacerbate these characteristics in developmental scale. We should further investigate the mechanisms by which screen exposure affects the neurodevelopment of children in the future.

### Strengths and limitations

This study used the ScreenQ, which is advocated by the AAP and reflects screen exposure in terms of “frequency of use, media content, and caregiver–child co-viewing” and is more authoritative than other scales.

This study did not delve into the potential mechanisms that might drive the correlation between screen exposure and neurodevelopmental deficits. Future research should integrate a multidisciplinary approach combining neuroscientific, psychological, and behavioral studies to unravel the complex interactions between screen exposure and autism. Longitudinal studies should be conducted in the future to track developmental changes over time.

In the future study, the sample size should continue to be expanded and it should be further explored whether there are other factors have mediating effect between screen exposure and neuropsychological development.

## Conclusion

ScreenQ can be used in the study of screen exposure in children with ASD. The higher the ScreenQ scores, the more severe the autistic symptoms tend to be, and the more delayed the development of children with ASD in the domains of personal-social, hearing-speech and eye-hand coordination. In addition, “frequency” has the greatest impact on the domains of personal social skills, hearing-speech, eye-hand coordination and performance of children with ASD.

## Data Availability

The datasets are available from the corresponding author on reasonable request.
